# Comparison of Efficacy of Coumarin and Chymotrypsin/Trypsin in Patients Undergoing Surgical Removal of Lower Third Molars: A Prospective Study

**DOI:** 10.7759/cureus.50750

**Published:** 2023-12-18

**Authors:** Gurmehr T Singh, Senthil Murugan P, Santhosh P Kumar, Murugesan Krishnan, Sibashish Khuntia

**Affiliations:** 1 Oral and Maxillofacial Surgery, Saveetha Dental College and Hospitals, Saveetha Institute of Medical and Technical Sciences, Saveetha University, Chennai, IND

**Keywords:** innovative technique, novel therapies, minor oral surgery, coumarin, chymotrypsin, trypsin, post-operative pain, post-operative swelling, quality of life, third molar surgery

## Abstract

Background

Third-molar surgeries are very commonly done by oral and maxillofacial surgeons. Pain and swelling that is associated with this procedure is a frequent reason for the patient’s discomfort and apprehension. There is a need to look for a drug that can substantially reduce postoperative swelling amongst the patients. Pain, swelling, and trismus are common complications that are encountered after third molar surgery. These complications have a major impact on the quality of life of patients undergoing minor surgical procedures.

Aim

The aim of this study was to compare the effectiveness of coumarin and trypsin/chymotrypsin in the reduction of postoperative sequelae for mandibular third molar surgeries.

Materials and methods

The research was carried out at Saveetha Dental College and Hospital in the Department of Oral and Maxillofacial Surgery. The study consisted of 50 individuals, 25 individuals received tablets of coumarin, and 25 individuals received tablets containing a combination of trypsin/chymotrypsin postoperatively. Patients were evaluated postoperatively for pain and swelling. Postoperative pain was measured on days one, three, and seven after surgery using a visual analog scale. The postoperative swelling was measured on postoperative days three and day seven via a four-point technique. Data were analyzed using IBM SPSS Statistics for Windows, Version 23 (Released 2015; IBM Corp., Armonk, New York, United States). P-values less than 0.05 were considered statistically significant. The independent samples t-test was used to compare the outcomes between the two groups.

Results

It was found that study participants in the trypsin/chymotrypsin group reported statistically significantly less pain postoperatively than participants receiving coumarin (p=0.001). There was more reduction in swelling postoperatively in patients who were given trypsin/chymotrypsin as compared to the participants who were given coumarin, and the results were statistically significant.

Conclusion

Based on the data obtained, it can be inferred that the trypsin/chymotrypsin combination was more effective in reducing postoperative sequelae like pain and swelling than coumarin in the mandibular third molar surgeries.

## Introduction

Impacted teeth in the oral cavity is a condition in which the teeth especially mandibular third molars are unable to erupt fully into occlusion in the oral cavity [1]. This is usually caused by the presence of a physical barrier which is usually nothing but other erupted teeth [2]. Completely impacted and unerupted teeth do not produce any disease usually except that sometimes, developmental cysts and neoplasms can develop from those teeth. The teeth that are partially impacted with their crowns partially erupted in the oral cavity, can cause dental caries or pericoronitis. In situations like these, the removal of the affected teeth is indicated [3].

Impacted mandibular third molars can be classified based on the direction of eruption, the relative position of the tooth in relation to the level of occlusal plane, the space available for the erupting tooth, and the amount of bone covering the tooth. The classification aids in evaluating the risk associated with the surgical removal of the teeth and the complications associated with them [4]. Impacted teeth can also be classified depending on the presence of infection or disease [5]. The diagnosis is usually made clinically. Radiographic investigations can also be taken along with clinical examination which includes orthopantomogram and cone beam computed tomogram (CBCT). The radiographic investigations usually aid in deciding the treatment plan. CBCT helps in locating the relative position of the vital structures associated with the tooth [6]. All the symptomatic impacted teeth can be initially treated with antibiotics and analgesics. Further operculectomy can be done. If the symptoms don’t subside, the surgical removal of the impacted teeth is carried out [7].

Trypsin/chymotrypsin works by breaking down proteins and increasing their absorption into the bloodstream. It improves blood flow and reduces inflammation, thus enhancing the rate of wound healing. Coumarin is known to prevent blood clot formation and it decreases inflammation as well as fluid retention. It combats inflammation and tissue edema (by reducing localized fluid retention). The aim of this study was to compare the effectiveness of coumarin and trypsin/chymotrypsin in the reduction of postoperative sequelae like pain and swelling for mandibular third molar surgeries.

## Materials and methods

Study design and setting

The participants for the study were recruited from the Department of Oral and Maxillofacial Surgery, Saveetha Dental College and Hospital, Chennai, India. The approval was obtained from the Institutional Human Ethics Committee, Saveetha Dental College (IHEC/SDC/OMFS-2206/23/254). The time period of the study was from December 2022 to April 2023. A total of 50 patients were included in this prospective study. Those patients who presented with an indication for surgical extraction of only the impacted mandibular molar teeth were recruited. The participants were informed, and written consent was obtained from each of them prior to the procedure. Using a random sampling technique, the samples were allotted into two groups. Each group had 25 samples in it. The allocation was done via opaque envelopes to prevent any bias.

Inclusion/exclusion criteria

Participants 18 years or older, presenting with class II and position A impacted mandibular third molar were included. Patients who were willing to report for regular follow-up and showed no signs of oral infection were included. Patients presenting with class I and class III impacted mandibular molars, with systemic diseases (like diabetes and hypertension), or those who were not willing to report for regular follow-up were excluded. Patients who showed signs of oral infection were also excluded from the study.

Surgical procedure

Under aseptic and sterile conditions, standard scrubbing was done following standard protocol. The patient’s intra-oral surgical site was prepared with betadine. About 2% lignocaine with adrenaline with 1:80,000 concentration was administered as an inferior alveolar, lingual, and long buccal nerve block on the side of the surgical procedure. Modified wards incision was given in all the patients and full thickness mucoperiosteal flap elevation was done. Bone guttering was carried out with copious amounts of saline irrigation. The tooth was carefully elevated and extracted in all the patients. The closure was done with a 3-0 silk suture and hemostasis was achieved without any untoward complication, in all the patients. Immediate postoperative medications like antibiotics and analgesics were given as per the standard protocol in all the patients. Double blinding was followed in this study, that is, both the surgeon and the patient were not aware of their allotted group and hence the bias was avoided. 0.12% chlorhexidine gluconate mouthwash was also prescribed in all patients for a week. Group A received trypsin/chymotrypsin combination tablets postoperatively for five days and group B received coumarin tablets postoperatively for five days.

Follow-up

**Figure 1:**
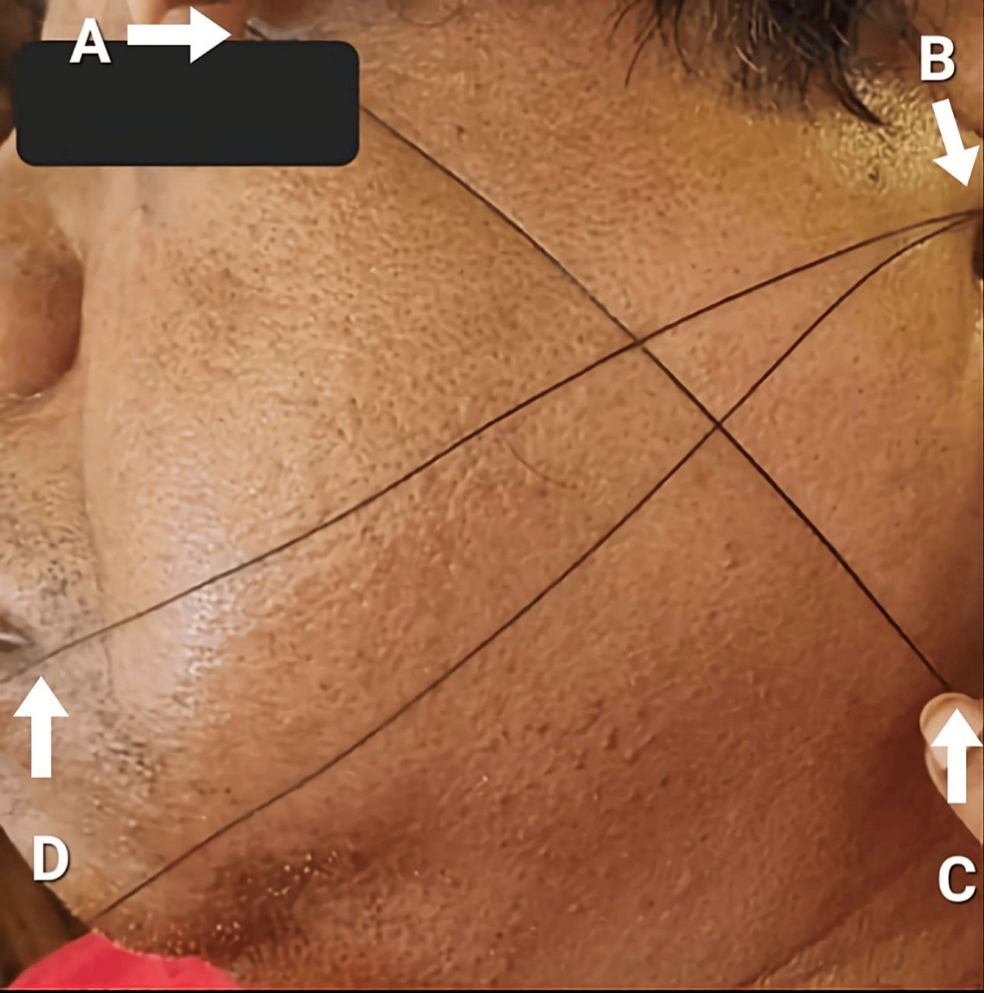
Facial measurement of swelling using the four-point technique A: Lateral canthus of the eye; B: Tragus of the ear; C: Angle of the mandible; D: Corner of the mouth

There were two parameters that were assessed in the postoperative time frame, that is, pain and swelling. These parameters were compared between the two groups. Facial measurements were done for each and every patient pre-operatively. After this, surgical extraction of the mandibular third molars was carried out. Following the procedure, pain among the participants was assessed via the 10-point visual analog scale where ‘0’ meant “no pain” and ‘10’ meant “worst pain imaginable”. The values were recorded on postoperative day one, day three, and day seven. Facial measurements were taken then postoperatively on day three and day seven via the four-point technique. As per this technique, four points were chosen, that is, A (lateral canthus of the eye), B (tragus of the ear), C (angle of the mandible), and D (corner of the mouth) (Figure 1). The horizontal measurement was done from the corner of the mouth to the tragus (from point B to D). The vertical measurement was done from the lateral canthus of the eye to the angle of the mandible (from point A to C). The mean of the two measurements was taken into consideration for the measurement of the facial swelling. This postoperative assessment was done by the same investigator who had carried out the surgical procedure on the patients.

Statistical analysis

The data was collected and analyzed using the IBM SPSS Statistics for Windows, Version 23 (Released 2015; IBM Corp., Armonk, New York, United States). Since the participants in group A were completely independent of the participants in group B, the unpaired student’s t-test was used to compare the independent groups. A p-value of less than 0.05 was considered to be significant. Shapiro-Wilk test was used to calculate the normality of the data.

## Results

The participants of the study were segregated into two groups, with 25 in each. All the patients were similar in their socio-demographic characteristics.

Pain measurement

Pain amongst the participants was assessed via the 10-point visual analog scale. The values were recorded on postoperative day one, day three, and day seven (Table 1).

**Table 1:** Comparison of pain scores among the two study groups * - statistically significant

Group statistics					
Visual analog scale scores at different timelines	Group	Number	Mean	Standard deviation	p-value
Postoperative day 1	TRYPSIN/CHYMOTRYPSIN	25	3.69	0.471	*0.001
	COUMARIN	25	5.20	0.402	
Postoperative day 3	TRYPSIN/CHYMOTRYPSIN	25	2.18	0.540	*0.001
	COUMARIN	25	3.22	0.384	
Postoperative day 7	TRYPSIN/CHYMOTRYPSIN	25	0.67	0.683	*0.001
	COUMARIN	25	1.92	0.450	

It was found that the study participants in the trypsin/chymotrypsin group reported less pain than participants receiving coumarin on postoperative days one, three, and seven, and the results were statistically significant (p=0.001).

Measurement of swelling

Facial measurements were taken pre-operatively and then postoperatively on day three and day seven. This was done using a four-point measurement scale. The mean of the two assessments was considered for assessment. The values obtained are depicted in the Table 2.

**Table 2:** Comparison of swelling values among the two study groups * - statistically significant

Group statistics					
Swelling measurements at different timelines	Group	Number	Mean	Standard deviation	p-value
Preoperative	TRYPSIN/CHYMOTRYPSIN	25	9.44	0.844	0.927
	COUMARIN	25	9.47	1.259	
Postoperative day 3	TRYPSIN/CHYMOTRYPSIN	25	9.75	0.851	*0.047
	COUMARIN	25	10.39	1.314	
Postoperative day 7	TRYPSIN/CHYMOTRYPSIN	25	9.47	0.848	*0.029
	COUMARIN	25	10.14	1.216	

It was found that there was less swelling in patients who were given trypsin/chymotrypsin as compared to the participants who were given coumarin on postoperative days three (p=0.047) and seven (p=0.029) and the results were statistically significant.

## Discussion

Surgical removal of impacted teeth especially mandibular third molars is one of the most common practices done by oral and maxillofacial surgeons. This surgical removal is often associated with postoperative complications. These complications are associated with the difficulty level of the surgical removal as well as the skills of the surgeon. The most common complications associated are pain, swelling, and trismus. Sometimes paresthesia can also occur due to nerve injury. The level of difficulty depends upon the angulation of the tooth, the depth and amount of bone covering the tooth, and also the vital structures associated. All these factors are responsible for increasing postoperative surgical complications [8]. It has been seen that the higher the difficulty level of the removal of the tooth, the more intraoperative time is taken to remove the tooth, and hence, for a longer period of time, the masticatory muscles and soft tissues are stretched. This prolonged stretching is responsible for the postoperative trismus or a limited mouth opening. Myositis involving the masticatory muscles is also a common complication, hence associated with the surgical procedure [9]. It is also seen that the more the postoperative pain, the more the trismus and decreased mouth opening. The more the manipulation of the soft tissues, or the higher the trauma associated with the soft tissues, the more the postoperative swelling and edema [10].

To control postoperative sequelae, steroids can be prescribed to patients. But their use is limited as it brings down the immune system. Steroids like dexamethasone are known to increase the blood sugar levels in patients with diabetes. Hence some alternate non-steroidal drugs need to be prescribed instead. In this study, two such drugs are chosen for comparison of their effectiveness, i.e., trypsin/chymotrypsin combination and coumarin. Trypsin/chymotrypsin is an enteric-coated tablet containing chymotrypsin and trypsin in a ratio of 1:6. Each tablet has the equivalence of 50,000 units of enzymatic activity [11].

Chymotrypsin is a digestive enzyme which is secreted by the pancreas into the duodenum. It aids in proteolysis and digestion. Without these enzymes, humans will not be able to convert large proteins into smaller ones and further into essential amino acids. Trypsin/chymotrypsin is neither an analgesic nor an antibiotic. Its main purpose is to control postoperative swelling in patients [12]. This drug should not be given to patients with glaucoma as it is known to aggravate the condition [13-15]. Trypsin/chymotrypsin works by breaking down proteins and increasing their absorption into the bloodstream. It improves the blood flow towards the wound or surgical site. Moreover, it also reduces inflammation in the region. Hence overall it aids in enhancing the rate of wound healing [16,17].

Coumarin is known to prevent blood clot formation. Apart from this, coumarin tablet decreases inflammation as well as fluid retention. It acts by inhibiting the hepatic enzyme glucose-6-phosphatase. It allows the blood to flow smoothly. It combats inflammation and tissue edema (by reducing localized fluid retention). Coumarin can also be used in lymphoedema and asthma and as a prophylactic for deep vein thrombosis [18]. Not much research has been conducted on the use of coumarin after minor surgical procedures. The drug has not yet been introduced in the field of oral and maxillofacial surgery. The purpose of this study is to introduce different drugs like coumarin in the field. However, coumarin has to be prescribed only to those patients who do not have any relevant medical or surgical history. Patients who already have bleeding tendencies like blood dyscrasias, vitamin K deficiency, patients with active ulceration in the gastrointestinal tract, or those patients who recently have undergone major surgery, cannot be prescribed coumarin. In all these conditions, the chance of uncontrollable bleeding drastically increases [19]. Common drugs that can interact with coumarin are thrombolytic agents like streptokinase, heparin, and aspirin; antibiotics like ciprofloxacin; and non-steroidal anti-inflammatory drugs like Ibuprofen or diclofenac [20]. Hence it should be prescribed after proper history taking and evaluation by the physician.

In this study, pain and swelling which are the two most common postoperative complications of third molar surgery were considered for evaluation. It was found that the patients who were given trypsin/chymotrypsin experienced less pain and had less swelling postoperatively as compared to the patients who were given coumarin. More research could be conducted to assess the long-term effects of each medication and to compare the effectiveness of these medications with other treatment strategies.

Limitations of the study

The study has a limited sample size. More studies need to be done with a larger sample size so that the results can be better analyzed and assessed.

## Conclusions

Based on the data obtained, it can be inferred that the trypsin/chymotrypsin combination was more effective in reducing postoperative sequelae like pain and swelling than coumarin in the mandibular third molar surgeries. Also, trypsin/chymotrypsin is an economical and easily available drug, hence can be used routinely after third molar surgeries.
